# Adherence to treatment in paediatric patients – results of the nationwide survey in Poland

**DOI:** 10.1186/s12887-020-02477-z

**Published:** 2021-01-06

**Authors:** Przemyslaw Kardas, Marek Dabrowa, Konrad Witkowski

**Affiliations:** 1 Department of Family Medicine, Medical University of Lodz, 60, Narutowicza St., 90-136, Lodz, Poland; 2 Department of Biopharmacy, Medical University of Lodz, Lodz, Poland

**Keywords:** Non-adherence, Patient compliance, Paediatrics, Surveys and questionnaires, Child, Clinical practice, Adolescent, Patient-centered care, Medication

## Abstract

**Background:**

Due to high prevalence, non-adherence to prescribed treatment seriously undermines the effectiveness of evidence-based therapies in paediatric patients. In order to change this negative scenario, physicians need to be aware of adherence problem, as well as of possible solutions. Unfortunately, full potential of adherence-targeting interventions is still underused in Poland. Therefore, the aim of this study was to assess the knowledge, attitudes and behaviours toward non-adherence in Polish paediatricians.

**Methods:**

An anonymous cross-sectional nationwide survey was conducted in the convenience sample of Polish doctors providing care to paediatric patients. The survey focused on the prevalence of non-adherence, its causes, and interventions employed. Primary studied parameter was perceived prevalence of non-adherence in paediatric patients. Reporting of this study adheres to STROBE guidelines.

**Results:**

One thousand and thirty-three responses were eligible for analysis. Vast majority of respondents were female (85.9%), most of them worked in primary care (90.6%). The respondents represented all 16 Polish Voivodeships, with the biggest number coming from the Mazowieckie Voivodeship (*n* = 144, 13.9%). Survey participants believed that on average 28.9% of paediatric patients were non-adherent to medication. More than half of the respondents (*n* = 548, 53.0%) were convinced that their own patients were more adherent than average. Duration of the professional practice strongly correlated with a lower perceived prevalence of non-adherence. Professionals with more than 40 years of practice believed that the percentage of non-adherent patients was <=20% particularly often (OR = 3.82 (95% CI 2.11–6.93) versus those up to 10 years in practice). Out of all respondents, they were also most often convinced that their own patients were more adherent than the general population (*P* < 0.01). Consequently, they underestimated the need for training in this area.

**Conclusions:**

Physicians taking care of Polish paediatric patients underestimated the prevalence of medication non-adherence and believed that this was a problem of other doctors. This optimistic bias was particularly pronounced in older doctors. These results identify important barriers toward improving patient adherence that are worth addressing in the pre- and post-graduate education of Polish physicians. They also put some light over the challenges that educational activities in this area may face.

## Background

Adherence to medication is the process by which patients take their medications as prescribed [1]. Consequently, various deviations from the prescribed treatment are collectively called “non-adherence”. Due to its widespread prevalence and serious consequences, WHO considers this phenomenon to be a “global problem of striking magnitude” [2]. As it has been observed many times, in the case of chronic diseases, at least 50% of patients do not use therapy in accordance with the instructions received from healthcare professionals. However, this percentage seems to be even higher in some countries. An international survey conducted in several European countries proved non-adherence to long-term treatment to be more prevalent in Poland than in all studied West European countries [3]. In a study involving over 63,000 Polish patients, non-adherence was found to be present in as many as 83.8% of them [4].

Adherence to medication is one of the basic preconditions of effective treatment. Taking medicines erroneously reduces the effectiveness of treatment and leads to the increase in healthcare costs [5]. Therefore, it is important to search for drivers of non-adherence, and to implement tailored evidence-based interventions that help to prevent and/or correct such a misbehaviour. Such interventions include, among the others, interventions based on treatment simplification, cognitive-educational interventions, behavioral-counseling interventions, social-psycho-affective interventions, technical equipment and reminders, monitoring feedback, and rewards [6]. Implementation of such interventions is especially important in case of the scenarios that are the most prevalent, and thus, the most important from the public health point of view.

In paediatric patients, non-adherence is common, and takes place both in chronic, as well as in acute conditions. It has been found to be prevalent across various areas of chronic paediatric care, including oncology [7], transplantology [8], nephrology [9], gastroenterology [10], and dermatology [11]. Contrary to the expectations, the same was true with acute conditions, which is best illustrated with short-term treatment with antibiotics. In a large sample of German children who were prescribed antibiotics due to various bacterial infection, overall adherence was 69.5% only [12]. Interestingly, even in case of children prescribed medications in an emergency department, over a quarter of them proved to be non-adherent [13]. Due to this high prevalence, non-adherence in paediatric care seriously undermines the effectiveness of treatment. Moreover, it relates to increased health care use in children and adolescents who have a chronic medical condition [14]. For all these reasons, non-adherence to medication in paediatric patients should be addressed in clinical care.

The role of physicians in ensuring optimal adherence of their patients is twofold. On one hand, it is important for them to take routine actions that may prevent non-adherence to therapeutic recommendations. These may include, among others, informing patients about the goals and methods of treatment in a way that has a real chance of building motivation for them to start therapy and continue it. On the other hand, it is also important to actively search for cases of non-adherence to therapeutic recommendations in order to implement appropriately selected corrective actions as soon as possible [15].

Despite the importance of this problem, there are relatively few studies on the knowledge of physicians and their actions to improve patient adherence in paediatric conditions. The research conducted in Poland showed that primary care physicians were not fully aware of the prevalence of non-adherence, and more importantly, they did not know interventions that may limit the scope of this problem [16]. Threfore, the purpose of this study was to assess the knowledge of physicians active in the field of child health in Poland regarding the phenomenon of non-adherence, as well as to assess their attitudes and behaviours toward this problem. Moreover, the study aimed to learn about the interventions they use to improve patient adherence to medication.

## Methods

### Study design, methods and participants

The study was conducted as a self-administered cross-sectional nationwide survey in the Polish physicians providing care to paediatric patients. It used the method of convenient sampling recruiting clinicians from all Polish Voivodeships (i.e. regions), who were listed in Aflofarm company database. The sampled cohort accounted for 31.9% of Polish paediatricians. The only inclusion criteria were belonging to the target cohort, and a free will to take part in the study, whereas lack of such a will constituted exclusion criterion. The survey questionnaire was made available in a digital version in a form of dedicated application. Forced answers to the survey questions were disabled. Due to cross-sectional pattern of the study, sample calculation was not applicable. In order to reduce social desirability and nonresponse bias, the survey was made anonymous.

### Study questionnaire

The survey measures included demographics, perceived prevalence of non-adherence in pediatric patients in general, knowledge, attitudes and behaviours toward this problem. Study questionnaire was built around these measures, partly based on the tool used in previous work [17]. First draft version of the survey tool has been subject of face validation by limited number of experts regarding aspects of content, construct and criterion validity. Next version of the tool was piloted in representants of the target group (10 in total) in order to fine-tune it. Analysis of the pilot results allowed for creating final version of the questionnaire, containing 6 items related to patient adherence in paediatric settings, and 6 questions about the characteristics of the respondents.

A phrase “not following the instructions that patients have received from their doctor regarding the use of medication” was used in the study questionnaire to describe non-adherence (see study questionnaire available in Appendix 1). Perceived prevalence of non-adherence in general pediatric population was assessed with percentage (objective measure), as well as in comparison to the patients of other doctors (contextual measure). Knowledge component investigated reasons for non-adherence in pediatric patients. Attitudes toward non-adherence was assessed with items assessing respondents’ ability to recognise non-adherence, as well as their need for additional training. Behavioural component checked for interventions employed by respondents for management of non-adherence. Due to the fact that at the moment, Polish physicians are not fully aware of adherence-enhancing interventions [16], and nationwide initiatives to change that scenario are lacking, several evidence-based, and easy to implement interventions identified so far were provided as answer options only [6]. Final version of the study tool is presented in Appendix 1.

### Ethical considerations

Before entering the survey questionnaire, survey participants received information regarding the aim and scope of the study, as well as intended use of collected data for scientific purposes only. Afterwards, they provided verbal informed consent for participation, as the surveying application did not provide any option for personal authentication. The survey was fully anonymized; therefore, according to the policy of Ethical Commission of Medical University of Lodz, the study was not subject to ethical approval.

### Data collection

The survey was made available in a digital version as a tablet-enabled application, provided to the target group by Aflofarm company staff. The survey fieldwork was conducted between August 28–September 19, 2019. Out of 1993 physicians invited to take part in the study, 1059 agreed (response rate 53.1%).

### Key variable definition

The percentage of pediatric patients not following the instructions they have received from their doctor regarding the use of medication has been defined as the answer to the question regarding perceived prevalence of non-adherence in paediatric patients.

### Data management, analysis and presentation

The data collected in the electronic survey application was transferred into the database created for this purpose and checked for completeness. Out of the total number of 1059 responses, 26 incomplete entries were removed from the final analysis, due to a lack of responses defining key parameter. Several responses lacking other variables were kept in the analysis.

In descriptive statistics, both original numbers and the percentages calculated out of the total number of analysed responses were presented, unless otherwise stated. The effect of potential modifiers of non-adherence-related parameters was assessed with univariable analysis, these modifiers including respondents’ characteristics (sex, specialisation, professional practice duration, primary workplace and Voivodeship). For the purpose of this analysis, professional practice duration (a continuous variable) was categorized into five categories: 1–10, 11–20, 21–30, 31–40 and > 40 years. Categorical variables were expressed as proportions and compared between relevant groups. For the analysis of the relationship between the observed results, the chi2 test (for qualitative variables) and the Kruskal-Wallis test (for quantitative variables) were used. A *P*-value of less than 0.05 was considered significant.

In addition to the descriptive analyses, multivariable analysis was performed with sex, specialisation, professional practice duration, type and Voivodeship of primary workplace accepted as independent variables, and perceived prevalence of non-adherence as a single dependent variable. In order to determine which of these factors, if any, were independently associated with perceived prevalence of non-adherence in our sample, a logistic regression was employed. This let the calculation of relevant odds ratios and 95% confidence intervals for chances that the perceived level of non-adherence was assessed as <=20%. For statistical calculations, Statistica 10 software (TIBCO Software Inc.) was used.

Study findings have been reported in compliance with STROBE checklist [18].

## Results

### Respondents’ characteristics

The analysis included responses provided by a total of 1033 physicians, the vast majority of whom (887, 85.9%) were women. More than ¾ of the respondents were paediatric specialists (*n* = 779, 75.4%). The vast majority of respondents (*n* = 936, 90.6%) indicated primary healthcare centres as their primary workplace. Most of respondents had over 20 years of work experience (*n* = 745, 72.1%), and the four oldest - over 50 years of work experience. The respondents represented all 16 Polish Voivodeships, with the largest number coming from the Mazowieckie Voivodeship (*n* = 144, 13.9%), and the smallest from the Lubuskie Voivodeship (*n* = 14, 1.4%). The detailed characteristics of the study participants are presented in Table 1.

**Table 1:** Study participants’ characteristics

Parameter	***N***	%
**Sex**		
Female	887	85.9
Male	118	11.4
Missing data	28	2.7
**Specialisation**		
Paediatrics	779	75.4
Family Medicine	183	17.7
Other	25	2.4
Missing data	46	4.5
**Professional practice duration (years)**		
0–10	109	10.6
11–20	177	17.1
21–30	363	35.1
31–40	294	28.5
> 40	88	8.5
Missing data	2	0.2
**Primary workplace**		
Primary care	936	90.6
Outpatient specialist clinic	54	5.2
Hospital	34	3.3
Missing data	9	0.9
**Voivodeship (region)**		
Dolnośląskie	78	7.6
Kujawsko-Pomorskie	58	5.6
Lubelskie	75	7.3
Lubuskie	14	1.4
Łódzkie	63	6.1
Małopolskie	96	9.3
Mazowieckie	144	13.9
Opolskie	24	2.3
Podkarpackie	39	3.8
Podlaskie	28	2.7
Pomorskie	43	4.2
Śląskie	100	9.7
Świętokrzyskie	51	4.9
Warmińsko-Mazurskie	66	6.4
Wielkopolskie	82	7.9
Zachodniopomorskie	48	4.6
Missing data	24	2.3
**Total**	**1033**	**100.0**

### Perceived prevalence of non-adherence

**Fig. 1:**
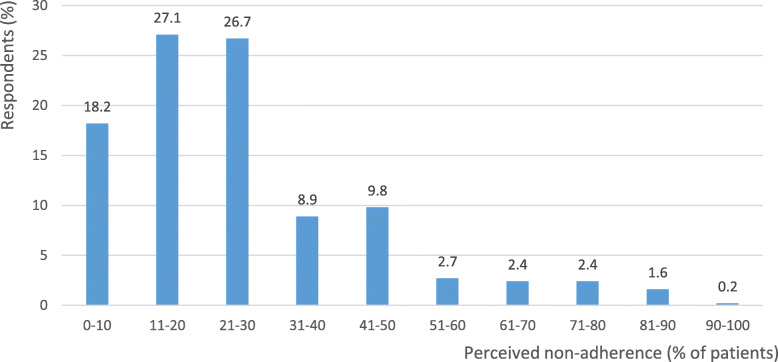
Distribution of the perceived non-adherence (percentages of paediatric patients who, according to respondents, did not adhere to medication)

Being asked about the percentage of all paediatric patients, who, while using medicines, did not comply with the instructions received from the doctor, respondents reported that this applied on average to 28.9% +/− 18.8% (mean +/− standard deviation, SD) of all paediatric patients. It is worth noting that the majority of respondents reported low values of this percentage, not exceeding 30% (in total 744 persons, 72.0%). The distribution of the percentages of paediatric patients who, according to respondents, did not adhere to medication is presented in Fig. 1.

**Fig. 2:**
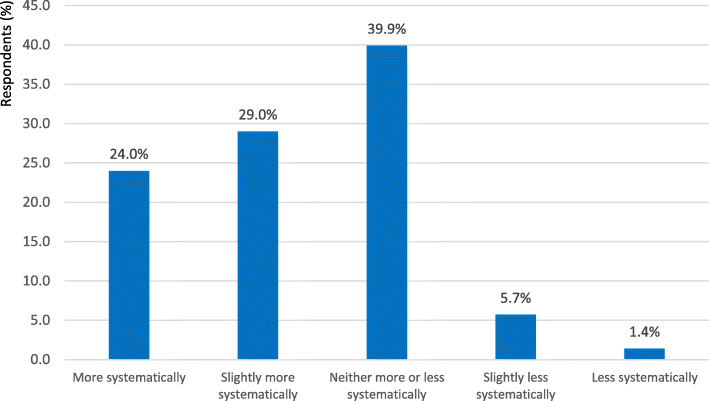
The degree to which the participants’ patients adhered to prescribed medication, as compared to all paediatric patients, according to survey participants

Respondents were also asked about how children whom they treat used their prescribed medicines, as compared to all paediatric patients. It is remarkable that as many as more than half of the respondents (*n* = 548, 53.0%) were convinced that the patients under their care were taking their drugs more or slightly more systematically than average (Fig. 2).

### Determinants of non-adherence

Respondents were asked to indicate the three most common reasons for non-adherence, among those presented to them. Results of this exercise are presented in Table 2. Forgetfulness was most often reported by nearly half of respondents (49.8%), followed by three other reasons provided by more or less 1/3 of respondents: lack of symptoms of the disease, frequent dosing schedule, and unacceptable dosage form of medicine. Poor access to medical facilities, and lack of trust in the doctor were provided least often (by 3.1, and 7.9% of respondents, respectively).

**Table 2:** Reasons for non-adherence to medication in paediatric patients and the interventions employed by study participants in order to improve adherence to medication in their paediatric patients

	***N***	%
**Reasons for non-adherence to medication**		
Forgetfulness	514	49.8
Lack of disease symptoms	380	36.8
Frequent dosing schedule	375	36.3
An unacceptable dosage form of medicine	364	35.2
Discouragement with long-term treatment	312	30.2
Misunderstanding of the disease and treatment goals	296	28.7
Drug adverse effects	288	27.9
High out-of-pocket drug costs	276	26.7
Lack of trust in the doctor	82	7.9
Poor access to medical facilities	32	3.1
**Interventions employed to improve adherence to medication**		
I educate child’s parents / guardians about the nature of the disease and the goals of treatment	753	72.9
I inform child’s parents / guardians about the consequences of non-adherence to medication	561	54.3
I detail the dosage regimen in a written form (on the sheet of paper / in a table)	552	53.4
I prescribe drugs administered less frequently	341	33.0
I schedule frequent check-ups	236	22.9
I prescribe inexpensive drugs	212	20.5
I try to use drugs with good organoleptic parameters	115	11.1
I choose drugs without adverse effects	82	7.9
I write out prescriptions for a longer period of time during one visit	54	5.2
I check the number of prescriptions issued in a given period of time	48	4.7

Notice: survey participants were asked to choose three most important reasons, and three interventions they employ most often, out of those listed; percentages calculated for the total *N* = 1.033 respondents

### Approach to non-adherence

Almost half of the respondents were convinced that they were able to recognize non-adherence to therapeutic recommendations in the majority of their patients, including 12.7% of doctors who thought that they could do it in almost every case, and further 36.9% who believed they could identify this behaviour in most cases. A slightly smaller percentage of respondents (40.8%) thought that they were able to recognize non-compliance with therapeutic recommendations in some cases, while only every tenth respondent believed that they could do it in a smaller part of their patients (of which 7.1% - in the minority of cases, and 2.5% - in almost no case).

Consequently, respondents were asked to indicate the three most common interventions they employed in order to help patient adherence (see Table 2). Nearly ¾ of respondents claimed that they educated the child’s parents / guardians about the nature of the disease and the goals of treatment (72.9%), whereas over half of respondents informed child’s parents / guardians about the consequences of unsystematic treatment (54.3%) and detailed the dosage regimen in a written form (53.4%). Interestingly, respondents much less often employed interventions based on the choice of drugs being more patient-friendly: less frequently administered (33.0%), inexpensive (20.5%), and free of adverse effects (7.9%).

Finally, respondents were asked about how well-informed they felt about the problem of patient non-adherence, and whether they felt the need for additional training. The vast majority of respondents felt very well informed, or rather well informed (20.8, and 45.4%, respectively), and definitely or rather did not feel the need for additional training in this area. The percentages of doctors assessing their competencies in a poorer way were definitely lower: 28.5% of respondents were considered as moderately well-informed, and perhaps in need of additional training, while being rather not very well-informed or very poorly informed was reported by 4.2 and 1.1% of respondents, respectively.

### Effect of respondents’ characteristics over their approach to non-adherence

Out of respondents’ characteristics, geographical location and the duration of the professional practice had a major impact on their approaches toward non-adherence in paediatric population.

**Fig. 3:**
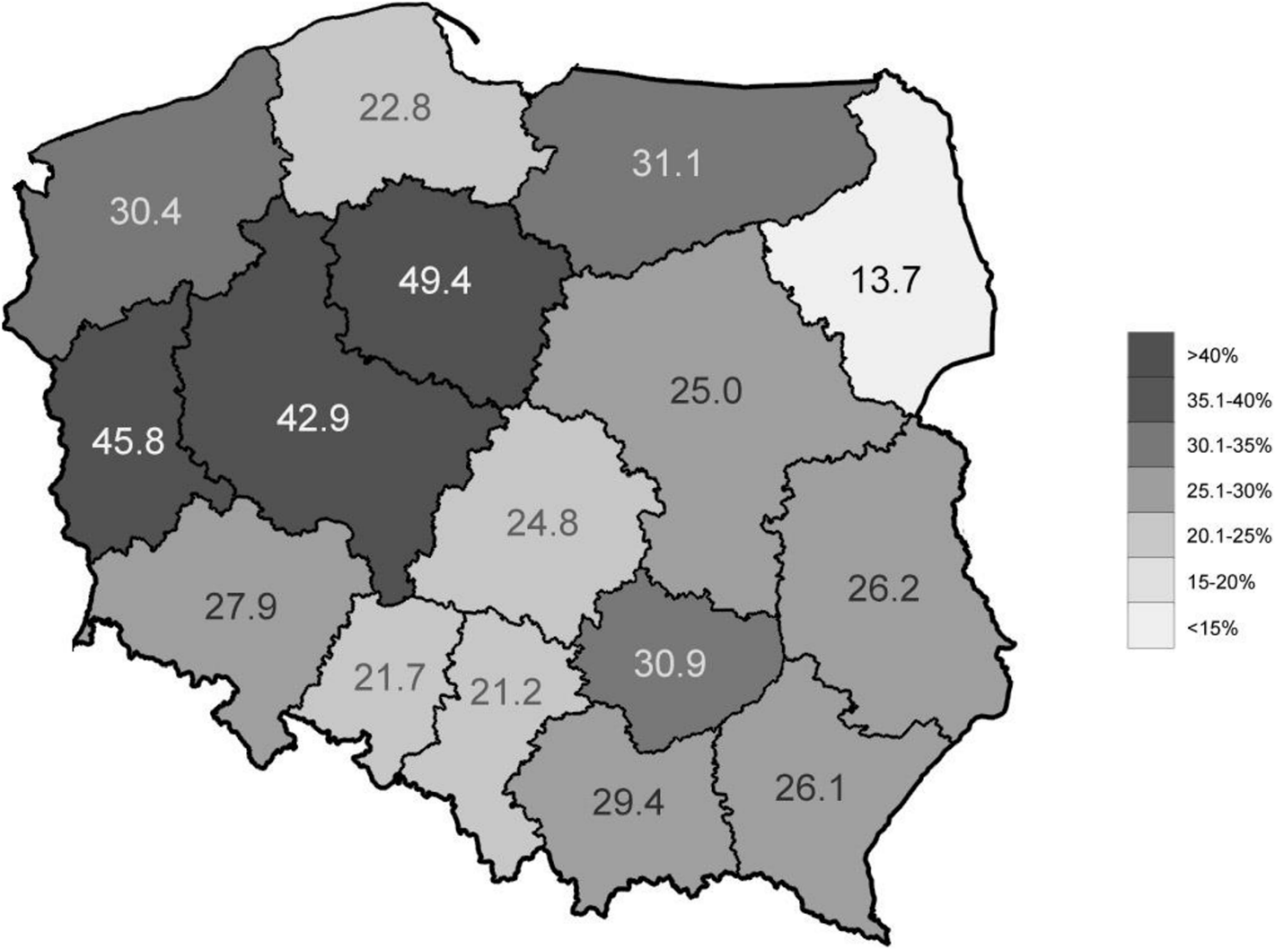
Average percentages of patients non-adherent to medication, as assessed by survey participants, by the Voivodeship of their workplace

**Fig. 4:**
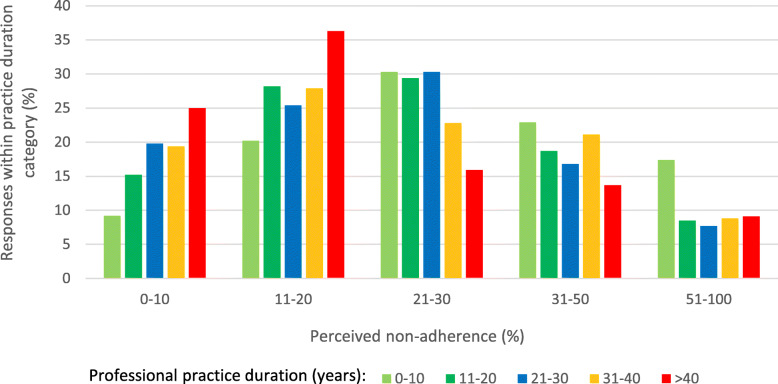
Percentage of patients non-adherent to medication, as assessed by survey participants (perceived non-adherence), by duration of their professional practice (in years)

**Fig. 5:**
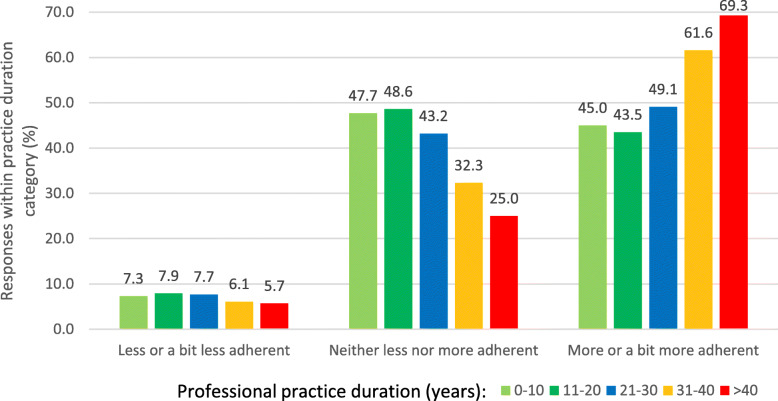
The degree to which the participants’ patients adhere to prescribed medication, as assessed by survey participants, by duration of their professional practice (in years)

The effect of geographical location on the perceived prevalence of paediatric patients’ non-adherence was clearly marked in univariable analysis, with a minimum value of 13.7%, on average, for the Podlaskie Voivodeship, and a maximum value of 49.4% for the Kujawsko-Pomorskie Voivodeship (*P* < 0.001, see Fig. 3). However, the duration of the professional practice consequently correlated with more optimistic bias toward non-adherence in paediatric patients: physicians with the longest duration of practice provided the lowest perceived percentages of non-adherence in paediatric patients (*P* < 0.01, Fig. 4), and much more often were convinced that their own patients were more adherent than the general population of patients (*P* < 0.01, Fig. 5). They also tended to believe most often that they were able to recognise non-adherence in all cases (*P* > 0.05), and finally, most often out of all physicians believed they were well-informed about the problem of non-adherence and did not need any additional education in that area (*P* < 0.01). Multivariable analysis confirmed the effect of duration of clinical practice and geographical location over perceived level of non-adherence. Of a special note is that the odds for low values of perceived nonadherence (range: 1–20%) increased with duration of professional practice, reaching a high of OR = 3.82 (95% CI 2.11–6.93) for professionals with more than 40 years of practice (Table 3).

**Table 3:** Effect of respondents’ characteristics on perceived prevalence of non-adherence. Multivariable logistic regression model for odds of perceived non-adherence to be assessed by survey participants as <=20% of pediatric patients

Variable	OR	95%CI	***P***
Sex			
Female	1.00	Reference group	
Male	0.78	0.53–1.16	*P* = 0.220
Specialisation			
Family Medicine	1.00	Reference group	
Paediatrics	0.83	0.58–1.2	*P* = 0.326
Other	0.67	0.28–1.61	*P* = 0.374
Professional practice duration (years)			
1–10	1.00	Reference group	1–10
11–20	1.85	1.11–3.08	*P* = 0.017
21–30	1.98	1.25–3.15	*P* = 0.004
31–40	2.16	1.35–3.46	*P* = 0.001
> 40	3.82	2.11–6.93	*P* = 0.000
Primary workplace			
Primary care	1.00	Reference group	
Outpatient specialist clinic	0.81	0.46–1.41	*P* = 0.447
Hospital	0.73	0.36–1.47	*P* = 0.371
Voivodeship (Region)			
Mazowieckie	1.00	Reference group	
Dolnośląskie	1.68	0.98–2.88	*P* = 0.058
Kujawsko-Pomorskie	0.30	0.14–0.63	*P* = 0.002
Lubelskie	1.19	0.69–2.06	*P* = 0.523
Lubuskie	0.24	0.05–1.11	*P* = 0.067
Łódzkie	2.05	1.14–3.68	*P* = 0.016
Małopolskie	1.22	0.74–2.01	*P* = 0.439
Opolskie	2.88	1.17–7.1	*P* = 0.021
Podkarpackie	1.00	0.73–1.37	*P* = 0.989
Podlaskie	6.63	2.4–18.27	P = 0.000
Pomorskie	2.43	1.22–4.84	*P* = 0.011
Śląskie	2.45	1.48–4.07	P = 0.001
Świętokrzyskie	0.86	0.45–1.63	*P* = 0.634
Warmińsko-Mazurskie	1.06	0.6–1.88	*P* = 0.838
Wielkopolskie	0.50	0.28–0.89	*P* = 0.018
Zachodniopomorskie	1.03	0.53–1.98	*P* = 0.932

*Legend*: *OR* odds ratio, *CI* confidence interval

The effect of the sort of primary workplace was marked, yet inconsistent. Primary care physicians were slightly less optimistic regarding the degree toward which their patients adhered to prescribed medication, as compared to hospital doctors, and ambulatory specialists (they believed their patients were more adherent, or slightly more adherent in 52.1% of cases, versus 63.7, and 66.7%, respectively, *P* > 0.05). At the same time, they felt better informed as to the problem of non-adherence than the other types of doctors. Ambulatory specialists much more often believed that non-adherence was caused by high out-of-pocket costs of the drugs (42.6%, versus 26.4% in case of primary care doctors, and 17.7% in case of hospital doctors, *P* < 0.05). They were also more optimistic regarding their ability to recognize the cases of non-adherence in their patients, as compared to the other types of doctors (e.g. 70.4% of them were convinced that they could disclose that in every or nearly every case, versus 49.0% of primary care doctors, and 41.2% of hospital doctors, *P* < 0.05).

Finally, neither respondents’ sex nor their specialization had a significant effect on the survey responses.

## Discussion

According to the authors’ knowledge, this was a first survey in Polish physicians which assessed approaches to patient adherence in their paediatric patients. With a nationwide coverage, this study proved that Polish physicians active in the field of child health were aware of the problem of non-adherence, but they seriously underestimated the actual prevalence of this phenomenon. Moreover, they demonstrated a significant optimistic bias, believing that their own patients adhered to the treatment much better than the average. At the same time, they overestimated their ability to recognize non-adherence in their patients. All these may discourage physicians from using available adherence targeting interventions, and lower the chances of pediatric patients to fully benefit from evidence-based therapies.

Adherence to medication in paediatric conditions is an issue of the utmost importance for individuals as well as for the entire population. Incorrect medication taking in these diseases is associated with deterioration of the quality of life of patients, increased morbidity and mortality, decreased productivity of their caregivers, as well as an increased use of health care services, and additional costs [15]. Moreover, in the case of anti-infective treatment, non-adherence may also lead to the emergence of resistant strains of pathogens [17].

In order to be ready to tackle this problem effectively, clinicians need to be aware of the phenomenon of non-adherence, and need to know the average level of non-adherence in their patients. A seminal WHO report popularised easy-to-remember statistics of 50% of patients being non-adherent to chronic treatments [2]. This, however, needs to be accepted with caution, as adherence varies across conditions, settings, and treatments. Recent systematic review of scientific literature identified 17 factors that influence adherence in paediatric oncology patients, falling into five major categories of patient/caregiver-related factors (e.g. disease and treatment perceptions), therapy-related factors (e.g. adverse effects of therapy, length and complexity of treatment), condition-related factors (e.g. poor prognosis), health system-related factors (e.g. poor healthcare provider communication and lack of supportive presence), and socioeconomic factors (e.g. financial difficulties) [19]. Similar exercise performed for chronic conditions in general population identified as many as 40 clusters of determinants of adherence [20].

Due to this, it is not possible to give one single number corresponding with the level of non-adherence in any patient group, including paediatric patients. Nevertheless, many studies in paediatric patients proved non-adherence to treatment of chronic diseases to even exceed the level of 50%. For example, adherence in paediatric patients with Attention Deficit/Hyperactivity Disorder was only 39.9% [21], whereas out of 2244 Spanish children prescribed antibiotics, only 46.5% adhered adequately [22]. Having this data in mind it would be fair to say that survey participants underestimated the overall prevalence of non-adherence in paediatric patients, providing the average number below 30%, and close to 1/5 of respondents believing that non-adherence occurs in up to 10% of the paediatric patients only. Respondents were also very optimistic regarding their ability to recognise the cases of non-adherence in their patients.

Similar tendencies have been observed in Poland beforehand in physicians treating chronic airways diseases, who seriously underestimated the prevalence of non-adherence. Moreover, almost 2/3 of them claimed that they could easily recognize non-adherence in their patients [23]. This, in fact, is far from reality, as studies consequently prove that healthcare professionals, even those knowing the patients for a long time, such as general practitioners, can predict potential non-adherence in their patients to the extent no better than resulting from chance [24, 25].

The optimistic bias of survey respondents was very clearly illustrated by the fact that as many as more than 50% of them believed that their own patients adhered better than average to prescribed treatment. This finding is consistent with the results of the European survey, in which healthcare professionals demonstrated optimistic bias about patients’ medication adherence, believing that their own patients were more likely to initiate and persist with treatment than other patients [26].

One possible explanation for these observations is that it is naturally difficult for physicians to accept that a significant proportion of their patients do not follow their recommendations. Daily observations seem to support these assumptions. After all, these patients report for follow-up visits and take next prescriptions. Hence the tendency to see this problem among other patients treated by other doctors only. Psychological background for this phenomenon has been named ‘availability heuristic’, and belongs to the biases typical for human thinking [27].

An unexpected finding of this study was the fact that this sort of bias was particularly pronounced in the oldest doctors. This may reflect more traditional, paternalistic approach to doctor-patient relationship. Over the time, this approach has been replaced by the new one, which accepts much more freedom in patients when it comes to defining and executing treatment plans. This is reflected by the shift that has taken place in relevant terminology. Namely, the old term of ‘compliance’, which evoked associations with paternalism and obedience, has been replaced by ‘adherence’, which accepts mutually agreed treatment plan to be a starting point for treatment [1].

Another unexpected finding of our study was a large variation of perceived levels of patient adherence across geographical locations, with much more realistic results observed to some regions in the west of Poland. To the authors’ knowledge, this phenomenon was observed for the first time. Therefore, there is no evidence to provide an explanation for this finding, and further studies are warranted to clarify the nature of this relationship.

Interestingly, survey participants much more often believed that the cases of non-adherence in paediatric patients were caused by patient/caregiver-, therapy- or condition-related factors (the three most often provided reasons were forgetfulness, lack of symptoms of the disease, and frequent dosing schedule), than the health system-related factors (lack of trust in the doctor was provided by 7.9%, and poor access to medical facilities by 3.1% of respondents only). In order to improve adherence, respondents used various forms of education and information rather than trying to make the treatment more patient-friendly (e.g. by assuring good organoleptic properties, positive pattern of adverse effects and low cost of drugs).

Of course, there is no single intervention able to secure full adherence in all the patients. On the contrary, it is hard to prioritise one intervention over the other due to their limited effectiveness [28]. Nevertheless, a comprehensive approach to increase treatment adherence in chronic paediatric conditions that has been proposed recently is worth considering. This approach is based on the three key elements: (1) a core approach to adherence promotion to be implemented by paediatric health care providers; (2) follow-up and ongoing management; and (3) tailoring and targeting specific more intensive family-centred interventions to children and adolescents who demonstrate clinically significant treatment non-adherence or risk for non-adherence [29]. Of practical usefulness could be a pre-consultation screening, allowing for identification of adherence problems. Recently, such a tool has been developed and found effective [30].

Finally, one should be aware that this study has its obvious limitations. The study was based on a survey, which is typically subject of the bias, such as social desirability and recall bias. Responses provided by survey participants to the questions related to the prevalence of non-adherence represented their speculations, rather than the results of any specific measurement, or real word statistics. Besides, these responses did not take into account the variation resulting from the different patterns of treatment adherence across various pediatric conditions (e.g. acute and chronic). Moreover, the method of selecting the study group does not guarantee its representativeness. However, according to the current data from National Medical Chamber, 15,053 doctors with a specialization in paediatrics are professionally active in Poland [31]. With the number of survey respondents exceeding 1000 we believe that the results collected are still very interesting. Their practical applicability comes with the fact that this study identifies important issues that are worth addressing in the pre- and post-graduate education of Polish doctors. According to our findings, special emphasis should be put to educating doctors with longer clinical practice, in whom the optimistic bias was particularly pronounced.

## Conclusions

Surveyed physicians active in the field of child health in Poland were aware of the phenomenon of non-adherence in paediatric patients, but they underestimated the actual prevalence of this phenomenon. They demonstrated a significant bias, believing that their own patients adhered to the treatment much better than the average. At the same time, they overestimated their ability to recognize non-adherence in their patients. All these may discourage physicians from using available adherence targeting interventions. Therefore, the results of this study indicate the issues that are worth addressing in the pre- and post-graduate education of Polish doctors dealing with childhood diseases. The primary target for such interventions are physicians with longer clinical practice, in whom the optimistic bias was particularly pronounced.

## Data Availability

The dataset supporting the conclusions of this article is available on written request to corresponding author.

## Appendix 1 - study questionnaire

Dear Doctor,

Please complete a short survey on the medication adherence in paediatric patients.

This survey is completely anonymous and is for scientific purposes only. The results of this study will be presented at the Polish Paediatric Society Congress 2019, as well as used in a scientific publication.

[Organizers].

1. In your opinion, what percentage of paediatric patients does not follow the instructions they have received from their doctor regarding the use of medication?
2. Compared to all paediatric patients, the children you take care of use the medication prescribed:

- More unsystematic
- Slightly more unsystematic
- On average, systematically
- A little more systematically
- More systematically

3. Why do not paediatric patients use prescribed medications according to doctor advise? Of the following, please select the 3 most important reasons, according to your opinion:

- Lack of disease symptoms
- Lack of trust in the doctor
- Frequent drug dosing schedule
- Adverse drug reactions
- Unacceptable dosage form of medicine
- Lack of understanding the disease and treatment goals
- Difficult access to medical facilities
- High drug costs
- Forgetfulness
- Discouragement with long-term treatment

4. Are you able to recognize non-adherence in a patient?

- In almost no case
- In a minority of cases
- In some cases
- In most cases
- In almost every case

5. Do you do anything to improve adherence of your patients? Please select a maximum of 3 actions most frequently undertaken by you:

- I educate the child’s parents / guardians about the nature of the disease and the goals of treatment
- I inform the child’s parents / guardians about the consequences of non-adherence to medication
- I plan frequent follow-up visits
- I prescribe less expensive medicines
- I prescribe drugs with less frequent dosing
- I check the number of prescriptions issued in a given period of time
- I try to use drugs with good organoleptic parameters
- I choose drugs without side effects
- I write prescriptions for a longer period of time during one visit
- I write the drug dosing scheme on a piece of paper/table

6. The problem of non-adherence to medication in paediatric patients is an issue in which you feel:

- Very well informed, and I definitely do not need additional training
- Rather well informed, and I do not really need additional training
- Moderately well informed, and maybe I need additional training
- Rather not very well informed, and I rather need additional training
- Very poorly informed, and I definitely need additional training

DEMOGRAPHIC DATA:

1. Age
2. Sex
3. Specialisation
4. Length of professional practice: ... (years)
5. Primary workplace:
  - Primary healthcare centre
  - Outpatient specialist clinic
  - Hospital
6. Voivodeship of the workplace

## Abbreviation

WHO: World Health Organisation
